# Effect of Whole-Body Cryotherapy on Antioxidant Systems in Experimental Rat Model

**DOI:** 10.1155/2017/8158702

**Published:** 2017-06-27

**Authors:** Bronisława Skrzep-Poloczek, Ewa Romuk, Bernadeta Wiśnowiska, Aleksander J. Owczarek, Piotr Choręza, Aleksander Sieroń, Ewa Birkner, Dominika Stygar

**Affiliations:** ^1^Department of Physiology, School of Medicine in Zabrze, Medical University of Silesia, Katowice, Poland; ^2^Department of Biochemistry, School of Medicine in Zabrze, Medical University of Silesia, Katowice, Poland; ^3^Department of Rehabilitation, 3rd Specialist Hospital in Rybnik, Rybnik, Poland; ^4^Department of Statistics, Department of Instrumental Analysis, School of Pharmacy with the Division of Laboratory Medicine in Sosnowiec, Medical University of Silesia, Katowice, Poland; ^5^Department of Clinical Internal Medicine, Angiology and Physical Medicine, School of Medicine in Zabrze, Medical University of Silesia, Katowice, Poland

## Abstract

**Background:**

The purpose of this study was to verify the effect of whole-body cryotherapy (WBC) in rats on their antioxidant systems, lipid peroxidation products, and their total oxidative status at different exposure times and temperatures.

**Methods:**

Antioxidants in serum, plasma, liver, and erythrocytes were evaluated in two study groups following 1 min of exposure to −60°C and −90°C, for 5 and 10 consecutive days.

**Results:**

WBC increased the activity of superoxide dismutase, catalase in the group subjected to 5 and 10 days exposure, −60°C. The glutathione S-transferase activity increased in the groups subjected to 10 days WBC sessions. Total antioxidant capacity increased after 5 and 10 days of 1 min WBC, −60°C; a decrease was observed at −90°C. A decreased level of erythrocyte malondialdehyde concentration was observed at −60°C after 5 and 10 days of cryostimulation. An increased concentration was measured at −90°C after 10 days, and increase of erythrocyte malondialdehyde concentration after 5 days, −90°C.

**Conclusions:**

To the best of our knowledge, this is the first research showing the effect of WBC in rats at different exposure times and temperatures. The effect of cryotherapy on enzymatic and nonenzymatic antioxidant systems was observed in the serum of animals exposed to a temperature of −60°C in comparison to control.

## 1. Introduction

Whole-body cryotherapy (WBC) is commonly used as a therapeutic method in rheumatic and inflammatory diseases and in the pain treatment of muscles, joints, soft tissues, and degenerative diseases of muscle spasticity [1–4]. During WBC, the subject is exposed to extreme cold temperatures for a short period of time. Current studies with WBC show a wide range of temperature and time of exposure applied during therapeutic sessions [5]. Physiological reactions to cryogenic temperatures include analgesic, anti-inflammatory, immune, circulatory, and hormonal changes. The detailed biochemical mechanism of the impact of whole-body cryotherapy on selected metabolic pathways is still not fully understood. Cryotherapy treatment is implemented in diseases that exhibit the participation of reactive oxygen's species (ROS). The main sources of ROS are mitochondria, oxidation of catecholamines, flavin, nucleotides, enzymes systems, and external factors such as xenobiotics, ionizing radiation, ultraviolet radiation, and many chemicals. Neutralization of oxidative stress is considered to be a key mechanism that can explain the positive impact of cryotherapy. The purpose of the present study was to verify the effect of whole-body cryotherapy (WBC) on the systematic antioxidant systems in rats in terms of exposure time and temperature. We hypothesized that WBC would have beneficial effects on enzymatic and nonenzymatic antioxidant system and lipid peroxidation.

## 2. Materials and Methods

### 2.1. Animals

Wistar FL rats (approximately 12-week-old males and weighing 300–470 g) were purchased from the Center of Experimental Medicine of the University of Silesia in Katowice, Poland. All study groups were housed and maintained in the same, controlled conditions, 22 ± 1°C, humidity 60% ± 5, and 12-hour light-dark cycles, with a standard diet and water ad libitum. The rat diet contained 24% protein, 4.9% fat, 7% crude ashes, 4.7% crude fiber, lysine (13.6 g/kg), calcium (12 g/kg), methionine (4.5 g/kg), and phosphorus (8.3 g/kg). The study was performed in compliance with international guidelines for the care and handling of laboratory animals. All animal experimental protocols were approved by the Ethics Committee of the Medical University of Silesia in Katowice, number 29/01.

### 2.2. Experimental Model

**Figure 1:**
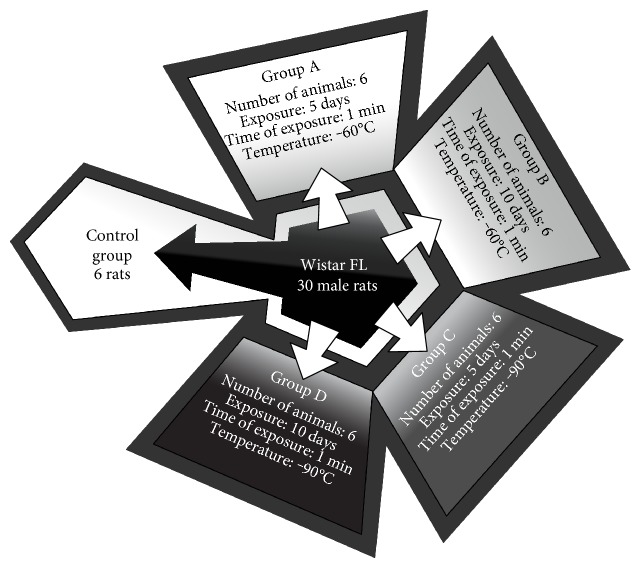
Scheme of the study groups.

All rats were randomly assigned to 5 study groups (*n* = 6, Figure 1). There was one control group. The control was treated in the same way as the experimental groups with one exception that it was not exposed to low temperatures. The end points to the control and experimental groups were the same in term of time and sampling. The animals from the experimental groups were exposed to either −60°C or −90°C for 1 minute in a cryogenic chamber for 5 and 10 consecutive days; henceforth, these exposure conditions are denoted −60/5, −60/10, −90/5, and −90/10, respectively (Figure 1). The values of temperature and time of exposure used in the study were assessed during a pilot study. Both temperature and exposure time were safe for animals and caused no harm or side effects.

### 2.3. Cryotherapy

The treatment of the animals with whole-body cryotherapy was performed within a cryochamber (cryochamber, type Wroclaw, Zagrobelny-Raczkowski-Strank, Wrocław, Poland). The cryochamber was divided into two compartments held at different temperatures (−60 and −90°C). The low temperature was maintained using liquid nitrogen circulating inside the chamber walls. The animals were weighed daily before WBC. The air in both chambers contained 22% oxygen and 78% nitrogen. The walls of the chamber were lined with multilayer thermal insulation for use at low temperatures. The cryochamber was equipped with three entrances. Each of the doors had a window, which allowed for the visual inspection of the treatment. The humidity and temperature were monitored by an operating system connected to the chamber. The rats were exposed to very low temperatures −60 and −90°C using the two-stage cryogenic chamber every day at the same time between 9 am and 10 am for 5 and 10 consecutive days (Figure 1). The physical activity and eating behavior were controlled daily. There were no clinical signs of complications after cryotherapy sessions in all subjected animals.

### 2.4. Sample Collection and Tissue Preparation

After 5- and 10-day treatment periods, anesthesia was induced and maintained using isoflurane 2% and oxygen flow at 2 L/min under spontaneous breathing. Blood (3–5 mL) samples were collected from the right ventricular via tubes with and without EDTA. For serum analysis, samples were collected after centrifugation at 4000 rpm for 10 minutes at 4°C; serum was subsequently snap frozen in liquid nitrogen and stored at −80°C until analysis. The erythrocytes were separated by centrifugation at 5000 rpm for 10 minutes at 4°C, washed three times with buffered NaCl solution (PBS: 0.01 mol phosphate buffer 0.14 mol NaCl, pH 7.4), chilled to 4°C, and finally snap frozen in liquid nitrogen and stored at −80°C until analysis. Before analysis, the erythrocytes were thawed, and the haemolysate of the washed red blood cells was diluted with distilled water and chilled to 4°C. After blood sampling, the liver was perfused with 0.9% NaCl and tissues were harvested and washed using normal saline (NaCl 0.9%). For oxidative stress markers, the tissue was homogenized (1 : 10 *w*/*v*) in 0.9% NaCl with homogenizer (Potter-Elvehjem PTFE pestle and glass tube, Sigma-Aldrich) and then centrifuged for 10 min employing 4000 rpm at 4°C and treated as one independent sample. Homogenates were snap frozen in liquid nitrogen and stored at −80°C until further analysis, for no longer than30 days. Samples protein concentration was determined by the Lowry methods [6].

### 2.5. Oxidative Stress Marker Analyses

#### 2.5.1. Oxidative Enzymes Analysis

An antioxidant system was analyzed by determining the activity of antioxidant enzymes: total superoxide dismutase activity (SOD, EC 1.15.1.1), catalase (CAT, EC 1.11.1.6), glutathione peroxidase (GPx, EC 1.11.1.9), glutathione transferase (GST, EC 2.5.1.18), and glutathione reductase (glutathione-disulfide reductase, GR, GSR, EC 1.8.1.7); nonenzymatic antioxidant system: the total antioxidant capacity (TAC), serum total bilirubin, and serum uric acid; and the lipid peroxidation by determining of malonic dialdehyde concentration.

#### 2.5.2. Superoxide Dismutase Analysis (SOD)

The total SOD activity in blood serum, haemolisates, and liver tissue was determined with the use of the spectrophotometric method by Oyanagui [7]. Enzyme activity was expressed as nitrite units (NU) per mL serum. One NU exhibits 50% inhibition of the formation of a nitrite ion under the method's condition [7].

#### 2.5.3. Catalase Activity (CAT)

The catalase activity in the haemolisates and liver homogenates was determinate by the Aebi methods [8].

#### 2.5.4. Glutathione Peroxidase Activity (GPx) (EC 1.11.1.9)

To measure the activity of glutathione peroxidase, the haemolisates and liver homogenates were incubated with GPx buffer (100 mM potassium phosphate with 1 mM EDTA pH 7.7), 40 mM sodium azide, GSH (diluted in 5% metaphosphoric acid), GR (GPx diluted in buffer), NADPH (diluted with sodium bicarbonate 5%), and 0.5 mM tert-butyl. The decay of NADPH concentration was evaluated for 10 minutes in a spectrophotometer, at 340 nm [9].

#### 2.5.5. Transferase Glutathione-S (GST) Activity (EC 2.5.1.18)

Transferase activity of glutathione-S (GST) (EC 2.5.1.18) in the haemolisates and liver homogenates were determined by the kinetic method of Habig and Jakoby [10].

#### 2.5.6. Glutathione Reductase Activity (GR) (EC 2.5.1.18)

GR assay kit to quantify the enzymatic activity in the haemolisates and liver homogenates was registered by a decrease in the concentration of NADPH in the samples using GR buffer (200 mM sodium phosphate pH 7.5, 6.3 mm EDTA) and kinetic reading was performed at a wavelength of 340 nm for 10 minutes [11].

#### 2.5.7. Lipid Peroxidation

Malondialdehyde (MDA) concentration was measured in samples of plasma, liver, and haemolysates according to the method described by Ohkawa et al. using the reaction with thiobarbituric acid with spectrophotometric detection employing 515 nm excitation and 552 nm emission wavelengths. MDA concentration was calculated from the standard curve, prepared from 1,1,3,3-tetraethoxypropane [12].

#### 2.5.8. Total Antioxidant Capacity (TAC)

Plasma TAC was measured using a commercial kit (Randox Co., England). The 2,2'azino-di-(3-ethylbenzothiazoline sulphonate) (ABTS) was incubated with a peroxidase (metmyoglobin) and hydrogen peroxide to produce the radical cation ABTS+, which has a relatively stable blue-green color, and measured at 600 nm. The suppression of the color was compared to the standard for TAC measurement assays (Trolox). The assay results are expressed as Trolox equivalent (mmol/L) [13].

#### 2.5.9. Uric Acid

Uric acid concentration was measured in the plasma using the colorimetric method (Roche, Cobas 6000e501).

### 2.6. Statistical Analysis

Statistical analysis was performed using STATISTICA 10.0 PL (StatSoft, Poland, Cracow) and StataSE 12.0 (StataCorp LP, TX, US). Statistical significance was set at a *p* value below 0.05. All tests were two tailed. The interval data were expressed as a mean value ± standard deviation in the case of normal distribution or as a median/interquartile range in the case of data with skewed or nonnormal distribution. Distribution of variables was evaluated by the Shapiro-Wilk test and the homogeneity of variances was assessed by the Levene test. For comparison of data, the one-way ANOVA analysis were used with Dunnett's post hoc test to the control group. Just in case of a skewed data distribution, a logarithmic transformation was done before analysis.

## 3. Results

**Figure 2:**
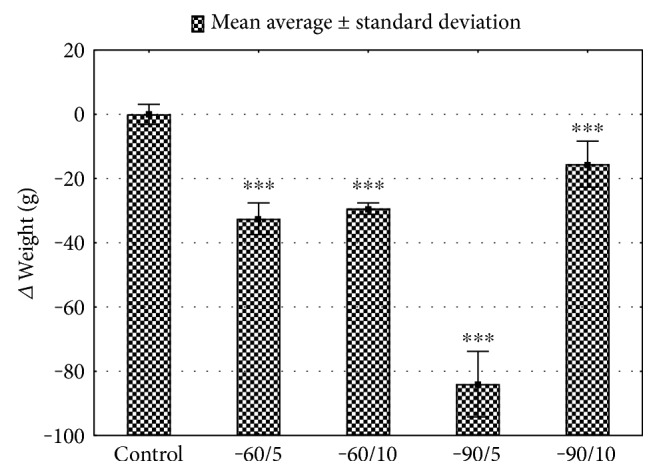
Δ weight of the study groups for various WBC treatments. Control: animals that did not undergo WBC treatment; group −60/5: 1 min of WBC stimulation, 5 days, −60°C; group −60/10: 1 min of WBC stimulation, 10 days, −60°C; group −90/5: 1 min of WBC stimulation, 5 days, −90°C; group −90/10: 1 min of WBC stimulation, 10 days, −90°C. Statistical significance was set at ^∗∗∗^*p* < 0.001, compared with control.

The basic biochemical parameters of the control and study groups are presented in Table 1. The plasma creatinine level significantly dropped after 10 days of exposure to −90°C (*p* < 0.001). The weight [g] of rats from the control group was significantly different from the studied group (*p* < 0.001, Figure 2). After 5 days of exposure to −90°C, the body mass of test rats was 22% lower compared with that of the control group.

### 3.1. Enzymatic Systems

**Figure 3:**
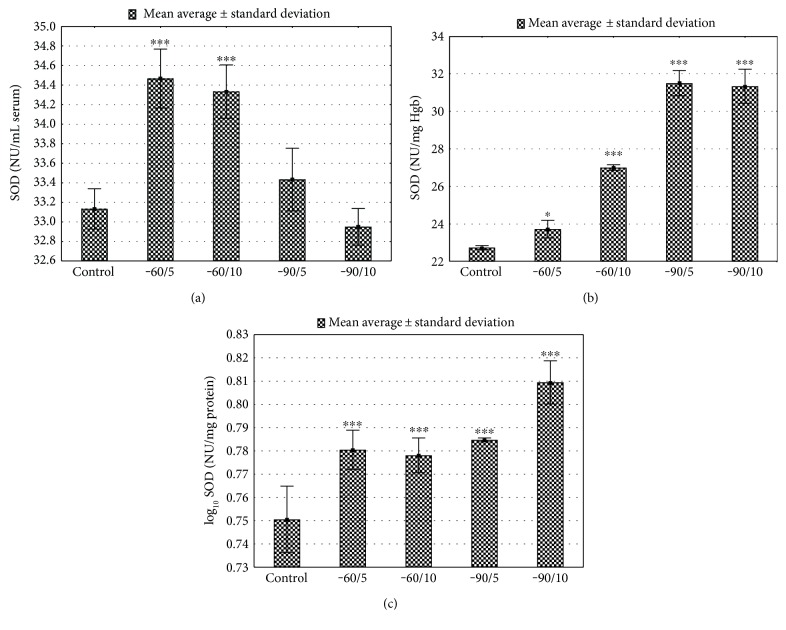
(a) Serum SOD activity for various WBC treatments. Control: animals that did not undergo WBC treatment; group −60/5: 1 min of WBC stimulation, 5 days, −60°C; group −60/10: 1 min of WBC stimulation, 10 days, −60°C; group −90/5: 1 min of WBC stimulation, 5 days, −90°C; group −90/10: 1 min of WBC stimulation, 10 days, −90°C. Statistical significance was set at ^∗∗∗^*p* < 0.001, compared with control. (b) Erythrocytes SOD activity after for various WBC treatments. Control: animals that did not undergo WBC treatment; group −60/5: 1 min of WBC stimulation, 5 days, −60°C; group −60/10: 1 min of WBC stimulation, 10 days, −60°C; group −90/5: 1 min of WBC stimulation, 5 days, −90°C; group −90/10: 1 min of WBC stimulation, 10 days, −90°C. Statistical significance was set at ^∗^*p* < 0.05 and ^∗∗∗^*p* < 0.001, compared with control. (c) Liver SOD activity for various WBC treatments. Control: animals that did not undergo WBC treatment; group −60/5: 1 min of WBC stimulation, 5 days, −60°C; group −60/10: 1 min of WBC stimulation, 10 days, −60°C; group −90/5: 1 min of WBC stimulation, 5 days, −90°C; group −90/10: 1 min of WBC stimulation, 10 days, −90°C. Statistical significance was set at ^∗∗∗^*p* < 0.001, compared with control.

**Figure 4:**
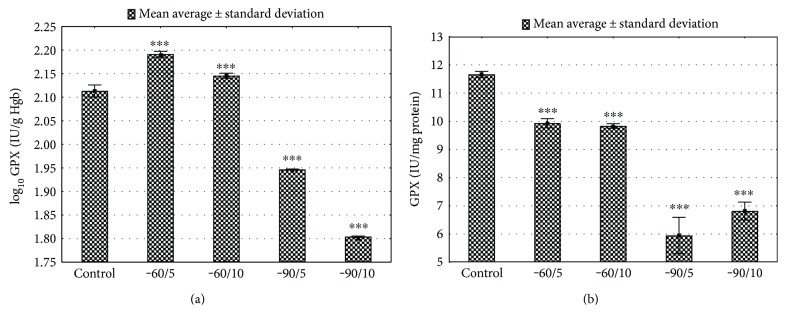
(a) Erythrocytes GPx activity for various WBC treatments. Control: animals that did not undergo WBC treatment; group −60/5: 1 min of WBC stimulation, 5 days, −60°C; group −60/10: 1 min of WBC stimulation, 10 days, −60°C; group −90/5: 1 min of WBC stimulation, 5 days, −90°C; group −90/10: 1 min of WBC stimulation, 10 days, −90°C. Statistical significance was set at ^∗∗∗^*p* < 0.001, compared with control. (b) Liver GPx activity for various WBC treatments. Control: animals that did not undergo WBC treatment; group −60/5: 1 min of WBC stimulation, 5 days, −60°C; group −60/10: 1 min of WBC stimulation, 10 days, −60°C; group −90/5: 1 min of WBC stimulation, 5 days, −90°C; group −90/10: 1 min of WBC stimulation, 10 days, −90°C. Statistical significance was set at ^∗∗∗^*p* < 0.001, compared with control.

**Figure 5:**
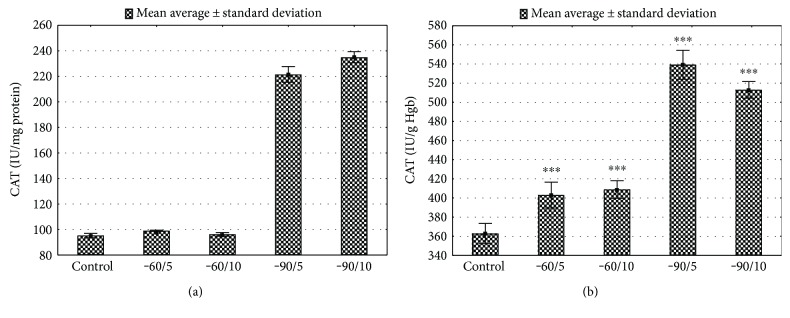
(a). Liver CAT activity for various WBC treatments. Control: animals that did not undergo WBC treatment; group −60/5: 1 min of WBC stimulation, 5 days, −60°C; group −60/10: 1 min of WBC stimulation, 10 days, −60°C; group −90/5: 1 min of WBC stimulation, 5 days, −90°C; group −90/10: 1 min of WBC stimulation, 10 days, −90°C. Statistical significance was set at ^∗∗∗^*p* < 0.001, compared with control. (b) Erythrocytes CAT activity for various WBC treatments. Control: animals that did not undergo WBC treatment; group −60/5: 1 min of WBC stimulation, 5 days, −60°C; group −60/10: 1 min of WBC stimulation, 10 days, −60°C; group −90/5: 1 min of WBC stimulation, 5 days, −90°C; group −90/10: 1 min of WBC stimulation, 10 days, −90°C. Statistical significance was set at ^∗∗∗^*p* < 0.001, compared with control.

The activity of SOD was assessed in serum, haemolysate (Hgb), and liver tissue. The serum SOD activity in the control group was significantly lower in comparison with the groups −60/5 and −60/10 (*p* < 0.001, Figures 3(a); basic serum biochemical parameters in Supplement Table 3 available online at https://doi.org/10.1155/2017/8158702). The total SOD activity in Hgb and liver tissue was significantly higher after cryotherapy in comparison to that in the control group (*p* < 0.05; *p* < 0.001 consecutively; Figures 3(b) and 3(c); basic serum biochemical parameters in Supplement Table 3). The activity of GPx measured in Hgb was significantly higher in −60/5 and −60/10 studied groups versus control (*p* < 0.001, Figures 4(a); Supplement Table 3). Erythrocytes GPx activity was lower in groups exposed to −90°C for 5 and 10 days (*p* < 0.001, Figure 4(a); Supplement Table 3). GPx activity in liver tissue was almost twice lower after 5 and 10 sessions at −90°C (*p* < 0.001, Figure 4(b); Supplement Table 3). The CAT activity in the liver and Hgb was significantly increased in the conditions of very low temperature (−90°C) when compared to the control (groups B and C, *p* < 0.001, Figures 5(a) and 5(b); Supplement Table 3). The CAT activity in the −60°C groups (groups A and B) represented an upward trend (*p* < 0.001, Figures 5(a) and 5(b); Supplement Table 3).

**Figure 6:**
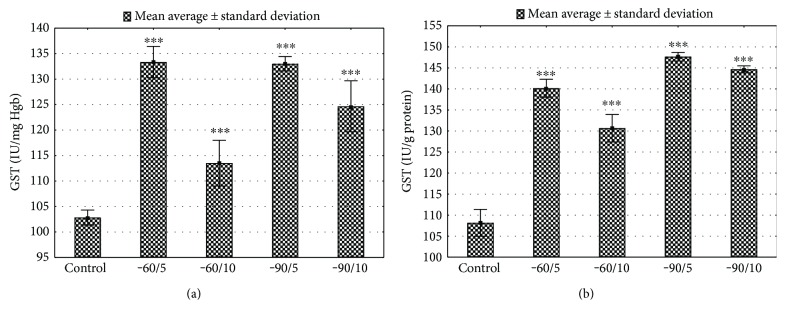
(a) Erythrocytes GST activity for various WBC treatments. Control: animals that did not undergo WBC treatment; group −60/5: 1 min of WBC stimulation, 5 days, −60°C; group −60/10: 1 min of WBC stimulation, 10 days, −60°C; group −90/5: 1 min of WBC stimulation, 5 days, −90°C; group −90/10: 1 min of WBC stimulation, 10 days, −90°C. Statistical significance was set at ^∗∗∗^*p* < 0.001, compared with control. (b) Liver GST activity for various WBC treatments. Control: animals that did not undergo WBC treatment; group −60/5: 1 min of WBC stimulation, 5 days, −60°C; group −60/10: 1 min of WBC stimulation, 10 days, −60°C; group −90/5: 1 min of WBC stimulation, 5 days, −90°C; group −90/10: 1 min of WBC stimulation, 10 days, −90°C. Statistical significance was set at ^∗∗∗^*p* < 0.001, compared with control.

**Figure 7:**
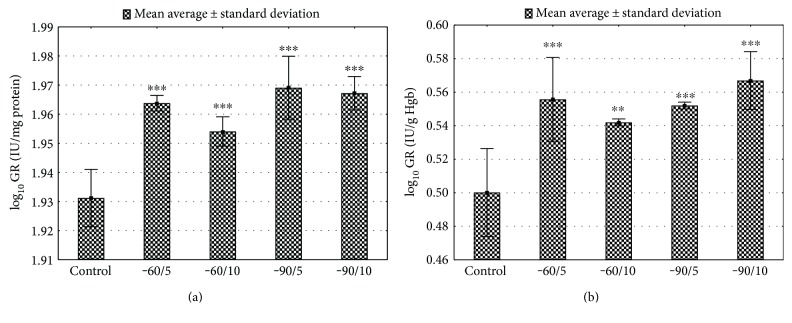
(a) Liver GR activity for various WBC treatments. Control: animals that did not undergo WBC treatment; group −60/5: 1 min of WBC stimulation, 5 days, −60°C; group −60/10: 1 min of WBC stimulation, 10 days, −60°C; group −90/5: 1 min of WBC stimulation, 5 days, −90°C; group −90/10: 1 min of WBC stimulation, 10 days, −90°C. Statistical significance was set at ^∗∗∗^*p* < 0.001, compared with control. (b) Erythrocytes GR for various WBC treatments. Control: animals that did not undergo WBC treatment; group −60/5: 1 min of WBC stimulation, 5 days, −60°C; group −60/10: 1 min of WBC stimulation, 10 days, −60°C; group −90/5: 1 min of WBC stimulation, 5 days, −90°C; group −90/10: 1 min of WBC stimulation, 10 days, −90°C. Statistical significance was set at ^∗∗^*p* < 0.01 and ^∗∗∗^*p* < 0.001, compared with control.

The short-term (5 days) and long-term (10 days) sessions of the exposure to the low range of temperatures increased the GST activity in erythrocytes and the liver (Figures 6(a) and 6(b), *p* < 0.001; Supplement Table 3). The whole-body cryotherapy stimulated the activity of GR both in the liver and in erythrocytes (*p* < 0.001, Figures 7(a) and 7(b); Supplement Table 3).

### 3.2. Nonenzymatic Antioxidant Systems

MDA plasma concentration was stimulated after 5 sessions of −90°C in relation to the control group (*p* < 0.001, Table 2). The lipid peroxidation measured by the concentration of MDA in erythrocytes was significantly suppressed in groups A and B versus the control group (*p* < 0.01, Table 2) and higher for the −90/10 group. The effect of oxidative stress expressed by the MDA concentration in liver tissue was observed in group −90/5, when compared to the control group (*p* < 0.001, Table 2). The whole-body cryotherapy influenced the TAC of plasma. TAC was significantly increased in −60°C (A and B groups, *p* > 0.001) and suppressed in −90°C (C and D groups, *p* > 0.001, *p* > 0.01) in comparison to the control. The serum uric acid concentration was significantly higher after long (10 days) exposure to −60°C (group B, *p* > 0.01) as well as short- and long-term (5 and 10 days) to −90°C (groups C and D, *p* > 0.001, *p* > 0.01) in comparison to the control group (Table 2).

## 4. Discussion

In this study, we have, for the first time, assessed the impact of four types of WBC treatments (−60/5, −60/10, −90/5, and −90/10) on the antioxidative systems of rats. It has been shown that, depending on the duration of exposure and the cryotemperature, WBC has a beneficial impact on mental health, pain, inflammatory response, and oxidative stress [5]. Two-week WBC (−100°C, 3-minute sessions) was shown to result in pain reduction for patients suffering from rheumatoid arthritis by inducing significant reduction of proinflammatory cytokines, blood concentration, IL-6, and TNF-*α*, as well as increased walking time and number of steps taken during a 50 m walking test [14, 15]. In this study, the body weight were reduced in all studied groups in comparison to that in the control group (Figure 2(); Supplement Table 3). The most severe body mass reduction was observed after exposure to −90°C for 5 days, which seem to be the less efficient among selected and applied therapeutic profiles. These changes can be interpreted as an effect of individual response. There was no change in the food consumption between the studied groups, and animals had unlimited access to food and water. The body mass reduction may be understood as a side effect of rapid adaptation to short-term severe stress caused by very low temperature. 10 days of exposure to low temperature may present more efficient and effective adaptation processes. Nevertheless, full understanding and explanation of this process in a more detailed study are required. Cold exposure is known to stimulate the oxidative stress and increase the antioxidative buffering capacity. Short-term exposure to very low temperatures is used in physical therapy to induce analgesic, anti-inflammatory, and antiswelling effects, as well as for reduction of muscle tension [16]. Circulatory adaptation to low temperature, the activity of GST total, lead to increased production of reactive oxygen species (ROS). Adaptation of animals to low temperature is associated with increased production of ROS by mitochondria and peroxisomes, which repeatedly increases the intensity of the process of fatty acids increased in the production of reactive oxygen species and oxidation [17–19]. Low temperature modifies the pro-oxidant-antioxidant homeostasis depending on the strength of the stimulus and the duration of exposure. The first line of cell protection against ROS is the enzymatic antioxidant systems (superoxide dismutase, glutathione peroxidase, and catalase) and nonenzymatic antioxidant systems (glutathione, ascorbate, and uric acid), which prevent lipid peroxidation and DNA damage [20]. The main line of defense against hydroxyl radicals is the dismutation process of superoxide anions (O_2_^−^) conducted by superoxide dismutase, disproportionation of H_2_O_2_ catalysed by CAT, and reduction with GPx [21]. The synthesis of SOD is stimulated by reduction of molecular oxygen, TNF, interleukins, and endotoxins and exposure to ionizing radiation, chemicals, and hypoxia [22, 23]. In this study, the total activity of SOD in the liver and Hgb was significantly elevated after 5- and 10-day WBC sessions at −60°C and −90°C when compared to controls. The increased total activity of SOD in the liver and Hgb may be an adaptive response of the cells to increased oxidative stress markers. Comparable results were presented for winter swimmers. In this studied group, the activity of enzymes reducing oxidative stress, such as SOD, CAT, and GPx, was increased in comparison to the control subjects [24–26]. Also, 20 WBC sessions (−110°C, 3 min each) increased the activity of superdioxide dismutase by about 43% in healthy men [16]. Demirbilek et al. showed that SOD activity in the gastric mucosa is increased under the influence of cold stress in comparison with the animals not exposed to low temperatures [27]. In the present study, total SOD activity measured in serum samples from animals exposed for 1 min, for 5 and 10 days, and to temperature at −60°C increased significantly when compared to the control group. The activity of this enzyme in −90/5 and −90/10 did not differ from the control group. We propose that the lower temperature −90°C caused more intensive production of ROS, which resulted in depletion of SOD blood plasma. Mila-Kierzenkowska et al. found a significant decrease in SOD and CAT serum activity in volleyball players immediately after WBC, which was applied in order to reduce the exercise-induced oxidative stress [28]. The activity of CAT measured in erythrocytes and livers of animals exposed to −90°C was significantly increased when compared with the control group. Increased CAT levels, together with reduced GPx activity in erythrocytes and liver cells, may be interpreted as the protective activity of CAT in the conditions of the intensive H_2_O_2_ production. CAT activity in the experimental groups exposed to −60°C showed an increasing trend, which confirms the mobilization of antioxidant enzyme system to stress-inducing factors. Lubkowska et al. presented similar results of CAT and GPx serum activity, which was found to be decreased 30 min after one session of WBC on healthy subjects [29]. In the erythrocytes and livers of animals subjected to a temperature of −90°C, the activity of GPx decreased in comparison to the control group. Similar studies conducted on 9 female kayakers who underwent ten-day cycle trainings preceded by 20 cryostimulation sessions at −120 up to −140°C for 3 min twice a day showed reduced activity of GPx and CAT. Reduced GPx activity in the liver of animals treated with cryotherapy at −90°C compared to the control group may be due to reduced biosynthesis of this enzyme or its inactivation. It is known that GPx is inhibited by superoxide radical O_2_ which is a SOD substrate. Hydrogen peroxide (H_2_O_2_) is an inhibitor of SOD, and superoxide ion inhibits GPx and CAT [29, 30]. This may explain the observed changes in the activity of SOD and GPx in this experimental model of cryotherapy. The increased activity of SOD is most likely induced by increased amounts of superoxide radicals produced during shivering thermogenesis. Superoxide radicals stimulate ischemia and reperfusion which causes hypoxia of endothelial cells, increases the energy demand, and intensifies mitochondrial metabolism [18]. All those changes result in a decline of GPx activity, leading to increased concentrations of H_2_O_2_ [31]. Reduction in GPx activity may be considered as the result of depletion of its antioxidant capacity. The reduced function of GPx may be compensated by the increased activity of GST, which in physiological conditions practically does not participate in free radical metabolism. After 5 and 10 sessions of whole-body cryotherapy at −60°C and −90°C, the activity of GST increased. Together with a higher total SOD activity, this may indicate mobilization of the antioxidant mechanisms of the cells. The increase in GST activity may be stimulated by intermediate products of lipid peroxidation, which increased due to the decrease in GPx activity. Moreover, GST is not inhibited by superoxide radicals. The glutathione plays an important role in protecting cells from the excessive oxidative stress. Oxidized glutathione is reduced by glutathione reductase (GR). The decline in the GSH : GSSG ratio is considered to be a sensitive indicator of the oxidative stress. WBC has been reported to either decline the GSH : GSSG ratio [16] or have no impact on it, depending on the time of exposure, number of sessions, and temperature declined. The increased activity of GR after cryogenic temperature exposure is correlated with GSH concentration, which is necessary for GST and GPx activity [32]. In the presented study, the increased activity of glutathione reductase, glutathione peroxidase, and superoxide dismutase after 5 and 10 cryosessions at −60°C shows that this temperature was significantly more beneficial for the induction of adaptive changes in the antioxidant system compared with lower temperatures (−90°C). In this study, we also observed changes in the plasma nonenzymatic antioxidant systems like uric acid, MDA, and TAC. Uric acid is considered to be one of the most important scavengers of reactive oxygen and nitrogen species [33, 34]. Plasma uric acid decreased after 5 WBC sessions, but significantly increased after 10 WBC sessions, which is consistent with the results presented by Lubkowska et al. [16]. This may confirm the primary role of uric acid in the antioxidant reactions and anti-inflammatory reactions in the body. The studies of WBC, combined with physical activity and exposure to different and more unstable conditions like winter swimming, show a rapid decrease in plasma uric acid levels [26]. This inconsistency may be caused by different mechanisms activated during contact with extremely cold water like shivering thermogenesis, more intensive heat loss, and production. More importantly, in this study, we observed a significant increase in plasma TAC after WBC sessions at −60°C and a decrease at −90°C compared with the control groups. Our data also showed a decrease and increase in erythrocyte MDA concentration, respectively, after 5 and 10 days of cryostimulation in −60°C and −90°C, and a significant increase for −90/5 in comparison with the control groups. This uneven trend may suggest that total antioxidant capacity and TBARS are not very good indicators for oxidative stress status in response to a very low temperature [16]. In contrast, the long-term exposure (3 min) to cryogenic temperatures result in an increased lipid peroxidation measured in erythrocytes with TAS and UA levels in plasma of healthy subjects [35].

## 5. Conclusions

WBC on rats exposed to −60°C and −90°C for 5 and 10 minutes in comparison to a control group not exposed to WBC were studied by its effect on the enzymatic and nonenzymatic antioxidant systems. WBC in rats leads to an increased activity of antioxidant enzymes in a group of animals exposed to a temperature of −60°C. The effect of cryotherapy on nonenzymatic antioxidant system was observed in the serum of animals exposed to a temperature of −60°C, which may suggest an elevation of the total concentration of antioxidants. The increase of lipid peroxidation processes in the plasma was observed only at −90°C. The temperature of −60°C influenced defense mechanisms against harmful lipid peroxidation. This effect is not observed during exposure at −90°C. These results may confirm the therapeutic effects of WBC in the treatment of diseases, which are connected with impaired functions of the antioxidant system of the body.

## Supplementary Material

Table. 3. Body weight of the studied groups, the activity of antioxidant enzymes: total superoxide dismutase (SOD) in serum and haemolisates (Hgb), glutathione peroxidase (GPx), catalase (CAT), glutathione-S-transferase (GST) as well as glutathione reductase (GR) in liver and haemolisates (Hgb) of rats after WBC.

## Figures and Tables

**Table 1:** Basic serum biochemical parameters. The concentration of plasma total protein and glucose and creatinine as well as urea acid in blood sera of rats after WBC. Values were expressed as a mean value ± standard deviation. Statistical significance was set at *p* < 0.05. All groups are compared to the control one.

Temperature (°C):		−60		−90		ANOVA	Multiple comparison with control			
Time:		5	10	5	10	*p*	A	B	C	D
	Control	Group A	Group B	Group C	Group D					
Plasma total protein [g/L]	64.33 ± 5.03	62.20 ± 4.65	63.18 ± 3.76	65.65 ± 2.06	65.65 ± 2.03	0.426	—	—	—	—
Glucose [mmol/L]	4.84 ± 0.22	5.15 ± 0.27	4.92 ± 0.24	5.35 ± 0.54	5.38 ± 0.64	0.118	—	—	—	—
Creatinine [*μ*mol/L]	43.45 ± 3.09	41.65 ± 2.09	44.01 ± 2.13	39.75 ± 3.25	37.08 ± 3.45	**<0.001**	0.690	0.991	0.107	**<0.001**
Urea [mmol/L]	6.31 ± 0.46	6.17 ± 0.31	6.16 ± 0.57	6.08 ± 0.14	6.28 ± 0.31	0.844	—	—	—	—

Control: animals that did not undergo WBC treatment; Group A −60/5: 1 min of WBC stimulation, 5 days, −60°C; Group B −60/10: 1 min of WBC stimulation, 10 days, −60°C; Group C −90/5: 1 min of WBC stimulation, 5 days, −90°C; Group D −90/10: 1 min of WBC stimulation, 10 days, −90°C.

**Table 2:** Nonenzymatic antioxidant system. The concentration of lipid peroxidation product: malondialdehyde (MDA) in plasma, haemolisates, and liver; total antioxidant capacity (TAC) in plasma and concentration of uric acid (UA) in serum of rats after WBC.

Temperature (°C):		−60		−90		ANOVA	Multiple comparison with control			
Time:		5	10	5	10	*p*	A	B	C	D
	Control	Group A	Group B	Group C	Group D					
MDA [mmol/L plasma]	2.49 (2.35–2.66)	2.63 (2.62–2.65)	2.63 (2.61–2.65)	4.12 (4.01–4.14)	4.15 (4.13–4.65)	**<0.001**	0.246	0.257	**<0.001**	**<0.001**
MDA [*μ*mol/g Hgb]	355.8 ± 11.0	319.5 ± 13.4	315.0 ± 23.6	349.0 ± 6.9	402.3 ± 24.4	**<0.001**	**<0.01**	**<0.01**	0.898	**<0.001**
MDA [*μ*mol/g protein]	330.8 ± 18.1	317.5 ± 12.5	339.2 ± 11.7	402.2 ± 6.7	315.3 ± 16.0	**<0.001**	0.285	0.672	**<0.001**	0.177
TAC [mmol/L]	2.03 ± 0.02	3.40 ± 0.21	2.90 ± 0.05	1.67 ± 0.09	1.76 ± 0.12	**<0.001**	**<0.001**	**<0.001**	**<0.001**	**<0.01**
UA [*μ*mol/L]	76.48 ± 2.92	67.82 ± 9.81	90.60 ± 8.82	55.40 ± 2.63	63.65 ± 2.36	**<0.001**	0.077	**<0.01**	**<0.001**	**<0.01**

Descriptive statistics and results of one-way analysis of variance. Values were expressed as a mean value ± standard deviation or as median/interquartile range in the case of data with skewed or nonnormal distribution. Statistical significance was set at *p* < 0.05. All groups are compared to the control one. Control: animals that did not undergo WBC treatment; Group A −60/5: 1 min of WBC stimulation, 5 days, −60°C; Group B −60/10: 1 min of WBC stimulation, 10 days, −60°C; Group C −90/5: 1 min of WBC stimulation, 5 days, −90°C; Group D −90/10: 1 min of WBC stimulation, 10 days, −90°C.
