# Supplementary material for: Effect of Whole-Body Cryotherapy on Antioxidant Systems in Experimental Rat Model
Source: Oxid Med Cell Longev. 2017 Jun 27;2017:8158702. doi: 10.1155/2017/8158702 (PMC5504965; doi:10.1155/2017/8158702)
Supplement: Supplementary file 1 — Table. 3. Body weight of the studied groups, the activity of antioxidant enzymes: total superoxide dismutase (SOD) in serum and haemolisates (Hgb), glutathione peroxidase (GPx), catalase (CAT), glutathione-S-transferase (GST) as well as glutathione reductase (GR) in liver and haemolisates (Hgb) of rats after WBC. [file 8158702.f1.docx]

Supplementary material.

Table. 3. Body weight of the studied groups, the activity of antioxidant enzymes: total superoxide dismutase (SOD) in serum and haemolisates (Hgb), glutathione peroxidase (GPx), catalase (CAT), glutathione-S-transferase (GST) as well as glutathione reductase (GR) in liver and haemolisates (Hgb) of rats after WBC.

| Temperature (°C): |  | -60 | | -90 | | ANOVA | Multiple comparison with control | | | |
| --- | --- | --- | --- | --- | --- | --- | --- | --- | --- | --- |
| Time: |  | 5 | 10 | 5 | 10 | p | A | B | C | D |
|  | Control | Group A | Group B | Group C | Group D |  |  |  |  |  |
| Δ Weight [g] | 0.0 ± 3.1 | -32.5 ± 4.9 | -29.3 ± 1.7 | -84.0 ± 10.2 | -15.5 ± 7.1 | **< 0.001** | **< 0.001** | **< 0.001** | **< 0.001** | **< 0.001** |
| Total SOD [NU/mL serum] | 33.13 ± 0.21 | 34.47 ± 0.30 | 34.33 ± 0.27 | 33.43 ± 0.32 | 32.95 ± 0.19 | **< 0.001** | **< 0.001** | **< 0.001** | 0.177 | 0.572 |
| Total SOD [NU/mg Hgb] | 22.75 ± 0.1 | 23.73 ± 0.47 | 27.00 ± 0.15 | 31.50 ± 0.67 | 31.33 ± 0.91 | **< 0.001** | **< 0.05** | **< 0.001** | **< 0.001** | **< 0.001** |
| SOD [NU/mg protein] | 5.60 (5.50 – 5.80) | 6.08 (6.05 – 6.09) | 6.02 (6.01 – 6.05) | 6.09 (6.08 – 6.10) | 6.45 (6.30 – 6.60) | **< 0.001** | **< 0.001** | **< 0.001** | **< 0.001** | **< 0.001** |
| GPX [IU/g Hbg] | 128.5 (127.0 – 134.0) | 154.0 (154.0 – 157.0) | 140.0 (139.0 – 141.0) | 88.4 (88.3 – 88.6) | 63.7 (63.5 – 93.9) | **< 0.001** | **< 0.001** | **< 0.001** | **< 0.001** | **< 0.001** |
| GPX [IU/mg protein] | 11.67 ± 0.1 | 9.93 ± 0.16 | 9.83 ± 0.08 | 5.95 ± 0.64 | 6.93 ± 0.32 | **< 0.001** | **< 0.001** | **< 0.001** | **< 0.001** | **< 0.001** |
| CAT [IU/g Hbg] | 362.8 ± 10.5 | 403.0 ± 13.5 | 408.8 ± 9.2 | 539.2 ± 15.1 | 513.0 ± 8.6 | **< 0.001** | **< 0.001** | **< 0.001** | **< 0.001** | **< 0.001** |
| CAT [IU/mg protein] | 95.3 ± 1.7 | 99.1 ± 0.5 | 96.2 ± 1.45 | 221.5 ± 6.2 | 235.0 ± 4.3 | **< 0.001** | 0.220 | 0.976 | **< 0.001** | **< 0.001** |
| GST [IU/mg Hbg] | 102.8 ± 1.5 | 133.3 ± 3.3 | 113.5 ± 4.5 | 133.0 ± 1.4 | 124.7 ± 5.0 | **< 0.001** | **< 0.001** | **< 0.001** | **< 0.001** | **< 0.001** |
| GST [IU/g protein] | 108.2 ± 3.2 | 140.2 ± 2.1 | 130.7 ± 3.3 | 147.7 ± 1.0 | 144.7 ± 0.8 | **< 0.001** | **< 0.001** | **< 0.001** | **< 0.001** | **< 0.001** |
| GR [IU/g Hbg] | 3.09 (3.05 – 3.15) | 3.64 (3.42 – 3.77) | 3.48 (3.47 – 3.50) | 3.57 (3.55 – 5.58) | 3.71 (3.56 – 3.76) | **< 0.001** | **< 0.001** | **< 0.01** | **< 0.001** | **< 0.001** |
| GR [IU/g protein] | 84.7 (84.5 – 85.0) | 91.7 (91.7 – 92.1) | 89.6 (89.1 – 90.7) | 93.2 (91.2 – 95.1) | 92.6 (92.1 – 93.4) | **< 0.001** | **< 0.001** | **< 0.001** | **< 0.001** | **< 0.001** |

Descriptive statistics and results of one-way analysis of variance. Values were expressed as a mean value ± standard deviation or as median/interquartile range in the case of data with skewed or non-normal distribution. Statistical significance was set at a *p* < 0.05.

Control: animals that did not undergo WBC treatment; Group A -60/5: 1 min of WBC stimulation, 5 days, -60 °C; Group B -60/10: 1 min of WBC stimulation, 10 days, -60 °C; Group C -90/5: 1 min of whole WBC stimulation, 5 days, -90 °C; Group D -90/10: 1 min of WBC stimulation, 10 days, -90 °C;
